# Prevalence of pelvic floor disorders in the Eastern Mediterranean region: A meta-analysis

**DOI:** 10.15537/smj.2023.44.2.20220510

**Published:** 2023-02

**Authors:** Hassan M. Elbiss, Wardah Rafaqat, Khalid S. Khan

**Affiliations:** *From the Department of Obstetrics and Gynecology (Elbiss), College of Medicine and Health Sciences, United Arab Emirates University, Al Ain, United Arab Emirates; from the Medical College (Rafaqat), Aga Khan University, Karachi, Pakistan; and from the Department of Preventive Medicine and Public Health (Khan), University of Granada, Granada, Spain.*

**Keywords:** pelvic floor disorder, pelvic organ prolapse, urinary incontinence, constipation, fecal incontinence

## Abstract

**Objectives:**

High prevalence of risk factors for pelvic floor disorders (PFD) in the Eastern Mediterranean may result in higher rates of prevalence of PFD in comparison to other regions. Despite individual studies, there are no clear statistics on the cumulative prevalence of PFDs in the East Mediterranean region. The aim of this study was to investigate the prevalence of PFDs in the Eastern Mediterranean region.

**Methods:**

A literature search without language restriction was conducted in PubMed, Cochrane database and Web of Science from 2016 to 01 June 2022. Cross-sectional and cohort studies that reported prevalence of urinary incontinence (UI), constipation, fecal incontinence (FI) and pelvic organ prolapse (POP) among women in the Eastern Mediterranean region were included. Study quality was assessed according to the Joanna Briggs Institute critical appraisal tool. Data were pooled and meta-analysed using a random effects model. PROSPERO: CRD42021283127

**Results:**

The search yielded 390 articles, from which 12 studies containing 9905 patients were included. Five studies were high quality and seven were low quality. The prevalence of POP (12 studies, 9905 participants), UI (5 studies, 2340 participants), constipation (4 studies, 2045 participants) and FI (1 study, 166 participants) was 39% (95% CI 21-57; I2 99.8%), 48% (95% CI 16-80; I2 99.7%), 39% (CI 17-60; I2 99.1%) and 14% (95% CI 9-20) respectively.

**Conclusion:**

In meta-analysis pooling studies of mixed quality, a high prevalence of PFDs was seen in the Eastern Mediterranean region.

**PROSPERO No.:** CRD42021283127

**P**elvic floor disorders (PFDs), including urinary incontinence (UI), fecal incontinence (FI) and pelvic organ prolapse (POP), are common debilitating conditions among women worldwide.^1^ They compromise physical, social, psychological and sexual function in women and thus, have a negative impact on their quality of life (QoL), worldwide including Saudi Arabia.^2,3^ Risk factors for PFDs including, high body mass index (BMI), number of vaginal deliveries, rate of fetal macrosomia, and lack of awareness of pelvic floor muscles exercise in the perinatal period are common in the Eastern Mediterranean region.^4-7^ Therefore, a higher prevalence of PFDs in comparison to other regions in the world may be anticipated.

Despite the presence of individual studies on the prevalence of PFDs in countries included in the Eastern Mediterranean region, an extensive literature search demonstrated that while there are several reviews on the prevalence of PFDs, no single evidence synthesis has been published to estimate of the disease prevalence in this region. A PubMed search for relevant reviews showed 312 citations of systematic reviews but none provided an evidence synthesis of studies covering the Eastern Mediterranean region. Thus, the objective of this meta-analysis was to summarize the available data on PFDs from studies conducted in the Eastern-Mediterranean region to estimate the regional prevalence of PFDs.

## Methods

This systematic review was carried out according to the PRISMA guidelines and the protocol was registered in PROSPERO prior to conducting the review.^8^ This cross-sectional and cohort studies that reported prevalence of UI, constipation, FI, and POP among women 18 years of age or above from countries located in the Eastern Mediterranean region were included. According to the definition provided by the World Health Organization (WHO), the following countries were considered part of the Eastern Mediterranean region: Afghanistan, Bahrain, Djibouti, Egypt, Iran, Islamic Republic of Iraq, Jordan, Kuwait, Lebanon, Libya, Morocco, Occupied Palestinian territory, Oman, Pakistan, Palestine, see Occupied Palestinian territory, Qatar, Saudi Arabia, Somalia, Sudan, Syrian Arab Republic, Tunisia, United Arab Emirates, Yemen.^9^

On June 2022 the databases of PubMed, Web of Science, and Cochrane Library were systematically searched. The following keywords were used: ‘epidemiology’, ‘occurrence’, ‘incidence’, ‘survey’, ‘frequency’, ‘rates or statistics’, ‘surveillance’, ‘pelvic floor dysfunction’, ‘pelvic floor defect’, ‘pelvic organ prolapse’, ‘uterine prolapse’, ‘genital prolapse’, ‘uterocele’, ‘rectocele’, ‘cystocele’, ‘rectal prolapse’, ‘bladder prolapse’, ‘Afghanistan’, ‘Bahrain’, ‘Djibouti’, ‘Egypt’, ‘Iran’, ‘Islamic Republic of Iraq’, ‘Jordan’, ‘Kuwait’, ‘Lebanon, ‘Libya’, ‘Morocco’, ‘Oman’, ‘Pakistan’, ‘Palestine’, ‘Qatar’, ‘Saudi Arabia’, ‘Somalia’, ‘Sudan’, ‘Syrian Arab Republic’, ‘Tunisia’, ‘United Arab Emirates’, ‘Yemen’, ‘Middle East’ and ‘Eastern Mediterranean’ (Appendix 1). The authors checked references of the included studies to find titles of additional possibly relevant articles. The title and abstract of the citations were reviewed independently by two reviewers. The full text of articles that either reviewer found relevant was acquired with the input of a library consultant. The full text was assessed independently by two reviewers for relevance. Any disagreements were settled by discussion and by consultation with a third reviewer.

Data were extracted independently by 2 reviewers. Disagreements were settled by using input from a third reviewer. The reviewers extracted data on the following variables were extracted: Study country and site, duration of recruitment, assessment method, and sample size. Primary outcome data consisted of number of women with POP, UI, constipation, FI, and total number of women assessed in each selected study.

Two reviewers used a published checklist to assess the methodological quality of the included studies.^10^ The scale assesses studies based on the following domains: representativeness of sampling frame, sampling method, a priori sample size calculation, valid method for outcome assessment, prospective study design, and response rate. Studies satisfying the criteria for at least 3/6 (50%) domains were considered high quality. The remaining studies were considered low quality. Disagreements between the two reviewers in judgement of the quality of the study were settled by discussion and consultation with a third reviewer. The conflict of interest and source of funding as reported by the studies included was also recorded.

### Data synthesis

Prevalence rates of PFDs were estimated with 95% confidence intervals (CI). Statistical heterogeneity was assessed graphically, using “forest” plots, and statistically, through the I^2^ statistic which gives a percentage of the variation across studies. Rate data were pooled and meta-analysed using a random effects model within Stata statistical software (Release V. 16. College Station, TX: StataCorp LLC (2019).

## Results

**Figure 1:**
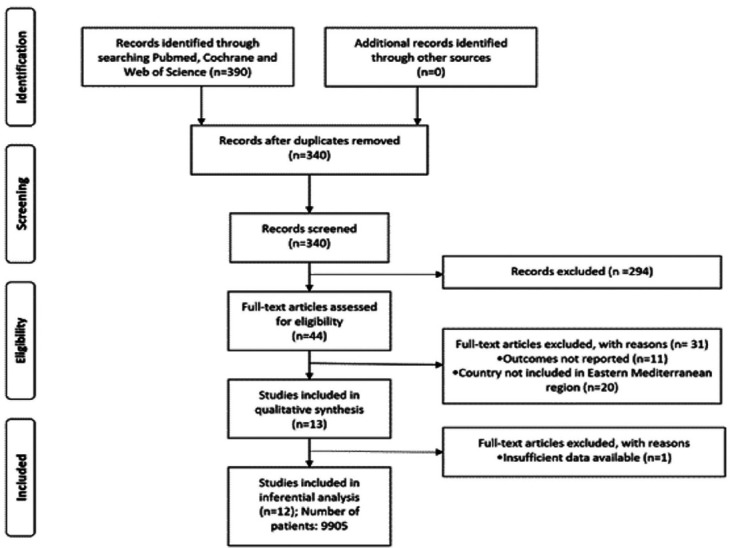
- Selection of studies in the meta-analysis on prevalence of pelvic floor dysfunction in Eastern Mediterranean region.

A total of 390 articles were identified from literature databases and through reference and citation searches. After exclusion of duplicates, there were 340 articles remaining, which were assessed for relevance by reviewing the title and abstract. Forty-four articles were found relevant and after the exclusion criteria were applied, thirteen articles were found relevant. One study, Dawood, et al. (2019) (12) did not report sample size and was not included in statistical analysis. Eventually, 12 studies were included in the meta-analysis (Figure 1).^4,11-22^

### Study characteristics and quality

A total of 9905 participants from five countries in the Eastern Mediterranean region were included. Data was collected from tertiary hospitals, primary health centers, and the community for a median of 12 months. The sampling methods used were convenience (n=9), random (n=4), and cluster sampling (n=1).^4,11-22^ One study used both random and convenience sampling.^16^ Patients were assessed using self-administered questionnaires (n=4), nurse-assisted questionnaires (n=2), and examination (n=5) (Table 1).^4,12-14,16-19,21,22^ Five studies^17,18,20-22^ were considered high quality and 8 studies^4,11-16,19^ were reported low quality according to the JBI critical appraisal tool (Table 2). Study quality assessment revealed concerns in a priori sample size estimation, selection of adequate sampling method and response rate (Table 2). Seven studies^13,16-18,20-22^ reported that funding was received, 10 studies^4,12-14,16-18,20-22^ reported no conflict of interest, and 3 studies^11,15,19^ did not comment on potential conflict.

### Prevalence of pelvic floor disorders

**Figure 2:**
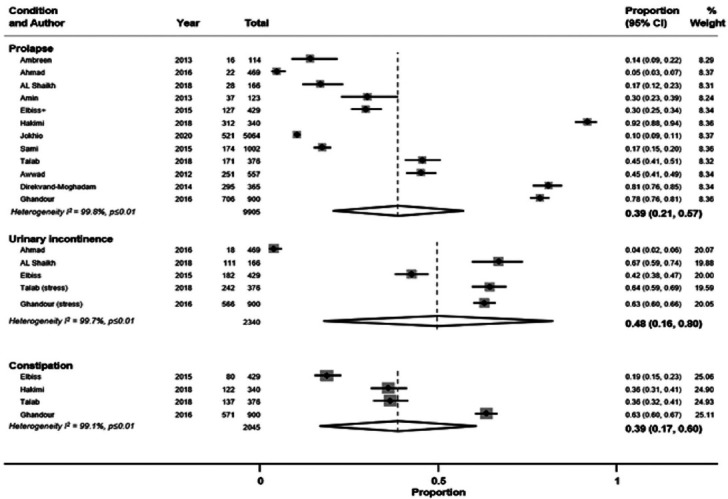
- Pooled prevalence of pelvic organ prolapse, urinary incontinence, and constipation in studies in the meta-analysis on prevalence of pelvic floor dysfunction in Eastern Mediterranean region.

**Figure 3:**
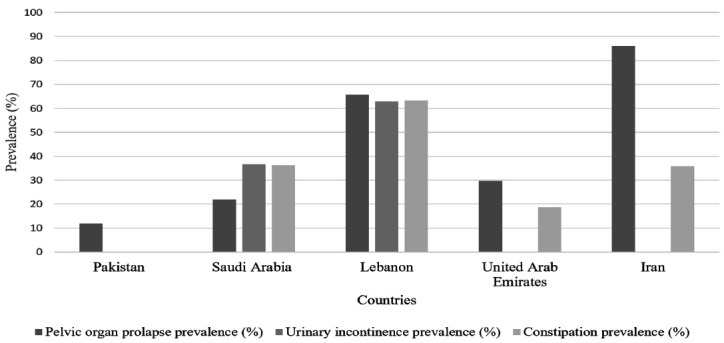
- Prevalence of pelvic organ prolapse, urinary incontinence, and constipation in studies according to the country in the meta-analysis on prevalence of pelvic floor dysfunction in Eastern Mediterranean region.

The prevalence rate of POP ranged from 5% to 92%, rate of UI ranged from 4% to 67%, and rate of constipation ranged from 19% to 63%. The prevalence rate of POP^4,12-21^ (12 studies, 9905 participants) was 39% (95% CI 21-57; I2 99.8%). The prevalence rate of UI 4,14,15,17,19 (5 studies, 2340 participants) was 48% (95% CI 16-80; I2 99.7%). The rate of constipation^4,13,14,21^ (4 studies, 2045 participants) was 39% (CI 17-60; I2 99.1%), and rate of FI_19_ (1 study, 166 participants) was 14% (95% CI 9-20) (Figures 2 & 3). Grade of prolapse was assessed in 6 studies (Table 3).

**Table 1:** - Characteristics of the selected studies in the meta-analysis on prevalence of pelvic floor dysfunction in Eastern Mediterranean region.

Study details	Country	Sampling strategy	Study site	Duration of recruitment	POP	Urinary incontinence	Fecal incontinence	Assessment method	Sample size
Ambreen et al^19^ (2013)	Pakistan (Lahore)	Random sampling	Fatima Memorial Hospital	12 months	Y	-	-	Self-administered questionnaire	114
Ahmad et al^16^ (2016)	Saudi Arabia (Riyadh)	Convenience and random sampling	Five health care centers	24 months	Y	Y	-	Nurse-administered questionnaire	469
Al Shaikh et al^18^ (2018)	Saudi Arabia	Convenience sampling	King Saudi University Hospital	12 months	Y	Y	Y	Self-administered questionnaires (PFDI, PFIQ and ICIQ-UI)	166
Amin et al^15^ (2013)	Pakistan	Convenience sampling	Mercy Teaching Hospital and Peshawar Medical College	24 months	Y	-	-	File review (patient history forms)	123
*Elbiss et al^14^ (2015)	United Arab Emirates (Al Ain)	Convenience sampling	3 family development centres	12 months	Y	Y	-	Self-administered questionnaire	429
Hakimi et al^21^ (2018)	Iran (Tabriz)	Random sampling	Health centres	6 months	Y	-	-	Physical examination	340
Jokhio et al^20^ (2020)	Pakistan	Random sampling	Primary Health Care Center	-	Y	-	-	Physical examination	5064
Sami et al^22^ (2015)	Pakistan (Karachi)	Multi-stage cluster sampling	Squatter settlements	12 months	Y	-	-	Nurse-administered questionnaire	1002
Talab et al^13^ (2018)	Saudi Arabia	Convenience sampling	King Fahad, Women’s Specialized Hospital	28 months	Y	Y	-	Physical examination	376
Awwad et al^17^ (2012)	Lebanon	Convenience sampling	Nabi sheet (rural community)	-	Y	-	-	Physical examination	557
Direkvand-Moghadam et al^12^ (2014)	Iran (Ilam province)	Convenience sampling	2 primary health centers	-	Y	-	-	Physical examination	365
Ghandour et al^4^ (2016)	Lebanon	Convenience sampling	University Medical Center Beirut	4 months	Y	Y	Y	Self administered questionnaire (PFBQ)	900
Dawood et al^11^ (2019)	Saudi Arabia	Convenience sampling	Primary Health Care centers	Y	Y	Y	-	-	-

^*^ Data extracted from 2 sources. Incontinence Questionnaire-Urinary Incontinence, POP-Q: Pelvic Organ Prolapse Quantification, PFBQ: Pelvic Floor Bother Questionnaire; - =No information provided; U and S: urinary and stress incontinence

**Table 2:** - Quality appraisal of included studies in the meta-analsyses of prevalence of pelvic floor dysfunction in the Eastern Mediterranean region.

Study details	Representative sampling frame	Adequate sampling method	Sample size calculation a priori	Valid methods for outcome assessment	Prospective study design	Response rate >90%	Overall quality (High/Low)^†^
Ambreen et al^20^ (2013)	N	Y	N	-	N	-	L
Ahmad et al^17^ (2016)	Y	N	N	Y	Y	-	L
Al Shaikh et al^19^ (2018)	Y	N	Y	Y	Y	-	H
Amin et al^16^ (2013)	N	N	N	-	N	-	L
*Elbiss et al^15^ (2015)	Y	N	N	N	Y	N	L
Hakimi et al^22^ (2018)	Y	Y	Y	Y	Y	-	H
Jokhio et al^21^ (2020)	Y	Y	-	Y	Y	Y	H
Sami et al^23^ (2015)	Y	Y	Y	-	Y	Y	H
Talab et al^14^ (2018)	Y	N	N	Y	Y	-	L
Awwad,et al^18^ (2012)	Y	Y	N	Y	Y	Y	H
Direkvand-Moghadam et al^13^ (2014)	Y	N	N	Y	N	-	L
Ghandour et al^4^ (2016)	Y	N	N	Y	Y	N	L
Dawood et al^12^ (2019)	-	Y	-	-	-	-	L

^*^ Data extracted from two sources, Sampling frame representative if target population described; Sampling method dequate if consecutive or random. Sample size calculation a priori if reported as such; Outcome assessment valid if measurement tool with a references;

^†^ High quality = criteria for atleast 4 quality ítems met, Y: yes, N: no, - : Not reported,

## Discussion

This systematic review of studies of mixed quality found through meta-analysis of heterogeneous results that in the Eastern Mediterranean region, around a little over a third of women suffered POP and constipation, nearly half of the women suffered UI, and a little over a tenth suffered FI.

We followed reported guidelines concerning systematic reviews.^23^ Our search was comprehensive and captured the geographical area of Eastern Mediterranean region with some gaps due to the scarcity of relevant prevalence studies in the region. A minority of the studies were high quality.^17,18,20-21^ The estimate of prevalence obtained was heterogeneous, with prevalence rates both very low and very high. This may be because of differences among population and clinical practice in the various countries.^5,24,25^ Risk factors for PFDs including, high BMI and high number of vaginal deliveries were reported in a majority of studies.^4,12-14,17,18,21^

A recent review and meta-analysis carried out in Ethiopia, a neighboring region, found out that nearly one fourth of the population suffered POP, whereas the rate of POP prevalence reported in our meta-analysis is almost twice this value.^26^ This difference might be due to the prevalence of high BMI prevalent in women of the Eastern Mediterranean region. Another review concerning PFD prevalence among women from LMICs reported the pooled prevalence for POP to be 15%, UI to be 30% and FI to be 8%. Our review observed the highest prevalence of UI followed by POP and FI among women from the Eastern Mediterranean region.^27^ Another review carried out to find out the prevalence of POP among United States racial population groups reported that white women (~11%) were most commonly affected followed by Hispanic (~6%), Black (~4%), and Asian American women (~3%).^29^ Our review provides comparative regional data adding to the existing information.^29-32^

**Table 2:** - Prevalence of grades of prolapse in the meta-analsyses of prevalence of pelvic floor dysfunction in the Middle Eastern Mediterranean Region.

Study details	Total (t)	No. of cases (n)	Grading tool	Grade of prolapse			
				Grade 1	Grade 2	Grade 3	Grade 4
Ambreen et al^20^ (2013)	114	16	-	0	10	6	0
Ahmad et al^17^ (2016)	469	22	International continence society staging system30	3	17	2	0
Amin et al^16^ (2013)	123	37	-	0	7	30	0
Jokhio et al^21^ (2020)	5064	521	Baden-walker classification system31	188	136	89	108
Awwad et al^18^ (2012)	557	251	POP-Q* system32	0	170	73	8
Direkvand-Moghadam et al^13^ (2014)	365	295	POP-Q system32	73	222	0	0

POP: pelvic organ prolapse

This is the first meta-analyses to summarize the prevalence of pelvic organ prolapse in the Eastern Mediterranean region and our findings merit consideration to inform policy and decision making. The findings of the meta-analysis show an extremely high prevalence of PFDs in this region and by doing so, it emphasizes the need for interventions for prevention of pelvic floor disorders in the Eastern Mediterranean region. While a few previous studies have looked into factors associated with increased prevalence of PFDs in the Eastern Mediterranean region, this study emphasizes the importance of increasing our understanding of the factors associated with increased prevalence of PFDs so that preventative measures can be taken especially, in high-risk women.

### Study limitations

This meta-analysis is characterized by some limitations. Firstly, the studies included in the meta-analysis were predominantly from Pakistan and Saudi Arabia. This limits its generalizability of this meta-analysis to the Eastern Mediterranean region. However, it also highlights the necessity for studies in other countries within the Eastern Mediterranean region to understand the epidemiology of this disease. Secondly, the technique for assessing pelvic floor disorders varied between studies. This contributes to the heterogeneity of the results of this meta-analysis. Due to the limited number of studies available, the authors were unable to perform a subgroup analysis according to the quality of the studies or evaluate for publication bias. These issues should be addressed in future evidence synthesis updates when more studies become available. The small number of studies per PFD did not allow for a meta-analysis for faecal incontinence and resulted in wide confidence intervals for POP and UI. This variation across point estimates of prevalence is a key limitation.

In conclusion, the prevalence of PFD in the Eastern Mediterranean region is high and to provide quality care to women, policy initiatives should prioritize resources towards this condition. Future research should explore reasons for heterogeneity and identify modifiable factors to prevent PFD. This first systematic review establishes the high prevalence of PFDs in the Eastern Mediterranean region.

## Appendix 1 - Search strategy and summary of search results from each database for the meta-analysis on prevalence of pelvic organ prolapse in the Eastern Mediterranean region.

((epidemiology) OR (occurrence) OR (incidence) OR (survey) OR (frequency) OR (rates or statistics) OR (surveillance)) AND ((pelvic floor dysfunction) OR (pelvic floor defect) OR (pelvic organ prolapse) OR (uterine prolapse) OR (genital prolapse) OR (uterocele) OR (rectocele) OR (cystocele) OR (rectal prolapse) OR (bladder prolapse))) AND ((Afghanistan) OR (Bahrain) OR (Djibouti) OR (Egypt) OR (Iran) OR (Islamic Republic of Iraq) OR (Jordan) OR (Kuwait) OR (Lebanon) OR (Libya) OR (Morocco) OR (Oman) OR (Pakistan) OR (Palestine) OR (Qatar) OR (Saudi Arabia) OR (Somalia) OR (Sudan) OR (Syrian Arab Republic) OR (Tunisia) OR (United Arab Emirates) OR (Yemen) OR (Middle East) OR (Eastern Mediterranean))

Date: 01 June 2022

The following are the search results from databases used:

**Table:**

PubMed	Keywords	Results
1	((epidemiology) OR (occurrence) OR (incidence) OR (survey) OR (frequency) OR (rates or statistics) OR (surveillance))	3, 850,555
2	((pelvic floor dysfunction) OR (pelvic floor defect) OR (pelvic organ prolapse) OR (uterine prolapse) OR (genital prolapse) OR (ureterocele)OR (rectocele) OR (cystocele) OR (rectal prolapse) OR (bladder prolapse))	10,990
3	((Afghanistan) OR (Bahrain) OR (Djibouti) OR (Egypt) OR (Iran) OR (Islamic Republic of Iraq) OR (Jordan) OR (Kuwait) OR (Lebanon) OR (Libya) OR (Morocco) OR (Oman) OR (Pakistan) OR (Palestine) OR (Qatar) OR (Saudi Arabia) OR (Somalia) OR (Sudan) OR (Syrian Arab Republic) OR (Tunisia) OR (United Arab Emirates) OR (Yemen) OR (Middle East) OR (Eastern Mediterranean))	540,633
4	((epidemiology) OR (occurrence) OR (incidence) OR (survey) OR (frequency) OR (rates or statistics) OR (surveillance)) AND ((pelvic floor dysfunction) OR (pelvic floor defect) OR (pelvic organ prolapse) OR (uterine prolapse) OR (genital prolapse) OR (uterocele)OR (rectocele) OR (cystocele) OR (rectal prolapse) OR (bladder prolapse))) AND ((Afghanistan) OR (Bahrain) OR (Djibouti) OR (Egypt) OR (Iran) OR (Islamic Republic of Iraq) OR (Jordan) OR (Kuwait) OR (Lebanon) OR (Libya) OR (Morocco) OR (Oman) OR (Pakistan) OR (Palestine) OR (Qatar) OR (Saudi Arabia) OR (Somalia) OR (Sudan) OR (Syrian Arab Republic) OR (Tunisia) OR (United Arab Emirates) OR (Yemen) OR (Middle East) OR (Eastern Mediterranean))	250

**Table:**

Web of Science	Keywords	Results
1	((epidemiology) OR (occurrence) OR (incidence) OR (survey) OR (frequency) OR (rates or statistics) OR (surveillance))	129,926,014
2	((pelvic floor dysfunction) OR (pelvic floor defect) OR (pelvic organ prolapse) OR (uterine prolapse) OR (genital prolapse) OR (ureterocele)OR (rectocele) OR (cystocele) OR (rectal prolapse) OR (bladder prolapse))	17,179
3	((Afghanistan) OR (Bahrain) OR (Djibouti) OR (Egypt) OR (Iran) OR (Islamic Republic of Iraq) OR (Jordan) OR (Kuwait) OR (Lebanon) OR (Libya) OR (Morocco) OR (Oman) OR (Pakistan) OR (Palestine) OR (Qatar) OR (Saudi Arabia) OR (Somalia) OR (Sudan) OR (Syrian Arab Republic) OR (Tunisia) OR (United Arab Emirates) OR (Yemen) OR (Middle East) OR (Eastern Mediterranean))	664, 907
4	((epidemiology) OR (occurrence) OR (incidence) OR (survey) OR (frequency) OR (rates or statistics) OR (surveillance)) AND ((pelvic floor dysfunction) OR (pelvic floor defect) OR (pelvic organ prolapse) OR (uterine prolapse) OR (genital prolapse) OR (uterocele) OR (rectocele) OR (cystocele) OR (rectal prolapse) OR (bladder prolapse))) AND ((Afghanistan) OR (Bahrain) OR (Djibouti) OR (Egypt) OR (Iran) OR (Islamic Republic of Iraq) OR (Jordan) OR (Kuwait) OR (Lebanon) OR (Libya) OR (Morocco) OR (Oman) OR (Pakistan) OR (Palestine) OR (Qatar) OR (Saudi Arabia) OR (Somalia) OR (Sudan) OR (Syrian Arab Republic) OR (Tunisia) OR (United Arab Emirates) OR (Yemen) OR (Middle East) OR (Eastern Mediterranean))	128

**Table:**

Cochrane Database of Systematic Reviews	Keywords	Results
1	((epidemiology) OR (occurrence) OR (incidence) OR (survey) OR (frequency) OR (rates or statistics) OR (surveillance))	539,113
2	((pelvic floor dysfunction) OR (pelvic floor defect) OR (pelvic organ prolapse) OR (uterine prolapse) OR (genital prolapse) OR (ureterocele))	2,356
3	((Afghanistan) OR (Bahrain) OR (Djibouti) OR (Egypt) OR (Iran) OR (Islamic Republic of Iraq) OR (Jordan) OR (Kuwait) OR (Lebanon) OR (Libya) OR (Morocco) OR (Oman) OR (Pakistan) OR (Palestine) OR (Qatar) OR (Saudi Arabia) OR (Somalia) OR (Sudan) OR (Syrian Arab Republic) OR (Tunisia) OR (United Arab Emirates) OR (Yemen) OR (Middle East) OR (Eastern Mediterranean))	37,445
4	((epidemiology) OR (occurrence) OR (incidence) OR (survey) OR (frequency) OR (rates or statistics) OR (surveillance)) AND ((pelvic floor dysfunction) OR (pelvic floor defect) OR (pelvic organ prolapse) OR (uterine prolapse) OR (genital prolapse) OR (uterocele)) AND ((Afghanistan) OR (Bahrain) OR (Djibouti) OR (Egypt) OR (Iran) OR (Islamic Republic of Iraq) OR (Jordan) OR (Kuwait) OR (Lebanon) OR (Libya) OR (Morocco) OR (Oman) OR (Pakistan) OR (Palestine) OR (Qatar) OR (Saudi Arabia) OR (Somalia) OR (Sudan) OR (Syrian Arab Republic) OR (Tunisia) OR (United Arab Emirates) OR (Yemen) OR (Middle East) OR (Eastern Mediterranean))	26
